# Higher-Order Language Dysfunctions in Individuals with Alcohol Use Disorder

**DOI:** 10.3390/jcm10184199

**Published:** 2021-09-16

**Authors:** Ewa Karabanowicz, Ernest Tyburski, Karol Karasiewicz, Adrianna Bober, Leszek Sagan, Monika Mak, Wioletta Radziwiłłowicz

**Affiliations:** 1 Institute of Psychology, University of Szczecin, 69 Krakowska Str., 71-017 Szczecin, Poland; ewa.karabanowicz@gmail.com (E.K.); karasiewiczkarol@gmail.com (K.K.); adrianaweronika@gmail.com (A.B.); 2 Institute of Psychology, SWPS University of Social Sciences and Humanities, 10 Kutrzeby Str., 61-719 Poznań, Poland; 3 Department of Neurosurgery, Pomeranian Medical University in Szczecin, 1 Unii Lubelskiej Str., 71-252 Szczecin, Poland; leszekm.sagan@gmail.com; 4 Department of Health Psychology, Pomeranian Medical University in Szczecin, 26 Broniewskiego Str., 71-457 Szczecin, Poland; monika.mak@gmail.com; 5 Institute of Psychology, University of Gdańsk, 4 Bażyńskiego Str., 80-309 Gdańsk, Poland; psywr@ug.edu.pl

**Keywords:** alcohol use disorder, AUD, higher-order language functions, executive functions, speed of processing, depression

## Abstract

Patients with alcohol use disorders (AUD) have difficulties with certain aspects of higher-order language functions (HOLF) but there is no data on a wide range of these functions in this group. Therefore, the aim of this study was to compare different aspects of HOLF in patients with AUD and healthy controls (HC). A total of 31 patients with AUD and 44 HC took part in the study. We assessed HOLF with the Right Hemisphere Language Battery (RHLB) and measured control variables: depression using the Patient Health Questionnaire (PHQ) as well as the speed of processing and executive functions with the Color Trails Test (CTT). Patients with AUD had lower results on nine RHLB tests. Moreover, AUD patients had higher scores on PHQ and longer reaction times on CTT. The differences in most RHLB results remained significant after co-varying the control variables. Patients with AUD have difficulties with making inferences from the text, understanding the meaning of individual words, metaphorical content, and prosody, which may impede the comprehension and production of discourse in which linguistic elements must be integrated with non-verbal cues and contextual information. These disturbances may impact various spheres of everyday life and negatively influence social, private, and professional functioning.

## 1. Introduction

Language and communication abilities related to higher-level processing refer to the use of language in particular contexts, the communication of intentions, goals, thoughts and emotions, as well as the use of symbols to communicate. To understand an utterance that is rooted in such abilities requires drawing subtle inferences based on knowledge of the speaker, language, and context. A given utterance may be interpreted in any number of ways, depending on, for example, the identity of the speaker, physical context, or the preceding conversation [1]. Among these abilities, we can distinguish lexical-semantic processes, prosody, discourse understanding, and production, as well as comprehension of indirect speech such as metaphors, irony, and humor. These can be described/defined as higher-order language functions (HOLF). One of the batteries, which assesses aspects of communications and language skills, is the Right Hemisphere Language Battery (RHLB [2]). This battery includes more tests compared to other similar batteries, allowing more general diagnoses to be made, and is newer than the Right Hemisphere Communication Battery (RHCB [3]. The Polish version has many modifications, which makes it more of a new Polish battery and not just an adaptation of the American version [4]. This battery is helpful in diagnosing quantitative (e.g., correct answers in tasks) and qualitative (e.g., errors and different aspects of discourse concerning polite phrases, properly during diagnosis, length of speech, gesticulation, and eye contact) language and communication disorders. The RHLB battery is a reliable and validated method and has been adopted in different countries [4,5,6]. Difficulties with HOLF can significantly lower effective communication in everyday situations and harm the relationships of people affected, leading to impaired social interactions or sometimes even to alienation from society due to inappropriate behavior [7]. These impairments can cause problems with understanding the intentions and emotions of other people, comprehension of non-literal expressions and can lead to inappropriate comments or omission of especially important information [8,9].

Both the linguistic and non-linguistic aspects of HOLF are supported by cortical areas in the left and right hemispheres, which has been confirmed by clinical and experimental data. These language processes appear to be linked with the temporoparietal, frontal, and cingular cortices; they are often bilateral in nature, although the right hemisphere appears to dominate [10,11,12,13,14]. In addition to the cortical brain regions, language processing also depends on the white matter bundles that connect the cortices [15,16].

Chronic consumption of alcohol leads to neurotoxicity, causing changes in grey and white matter structure, as well as in the activation and functional connectivity of different brain areas [17,18]. The results of some recent meta-analyses [19,20,21,22] showed a reduction of volume in various cortical (e.g., superior frontal gyrus, prefrontal cortices, superior temporal gyrus) and subcortical (e.g., striatum, hippocampus, lenticular nuclei) regions of both hemispheres in alcohol use disorder (AUD) patients. Other meta-analytic findings suggest disturbances of white matter and bundles that connect cortical areas in the left and right hemispheres [23,24,25]. The meta-analysis of Quaglieri et al. [26] showed changes in the activation of different bilateral areas in the brain (e.g., frontal gyrus, left middle cingulate cortex, and inferior portion of the left putamen) in AUD patients. Such extensive changes in the brain can be related to HOLF impairments in patients with AUD.

There is very little research into particular aspects of HOLF in AUD patients [27] and most existing research concerns emotional processing [28,29,30]. Moreover, there is a lack of studies that simultaneously measure a wide range of HOLF in this clinical group. Some previous studies have shown that people with AUD have problems with the emotional decoding of affective prosody, expressing emotions, experiencing empathy, and recognition of the mental states of others [31]. Affective prosody deficits (a non-linguistic feature of language that conveys emotions and attitudes during discourse) were evident, especially in ambiguous situations, where there were no additional cues to interpret emotional prosody [32]. Impaired processing of emotion conveyed by music has also been observed [33]. The study of Maurage et al. [34] had people with AUD assess the intensity and type of emotions in various stimuli. They found that AUD patients had difficulties in emotional prosody. In another study [35], AUD patients generated significantly fewer socially sensitive and practically effective solutions for problematic interpersonal situations than a healthy control (HC) group. Furthermore, patients performed significantly worse when asked to select the best alternative from a list of alternative scenarios containing sarcastic remarks and had significantly more problems interpreting sarcastic remarks in difficult interpersonal situations.

Individuals with AUD reveal problems processing figurative content, such as understanding humor and detecting irony [28,30]. However, there is a lack of data on metaphor processing in people with substance use disorders. Humor plays a very important role in social interaction. It is associated with wellbeing, psychological health, and has a beneficial effect on immune system activity [36,37]. The use of this competence requires the involvement of three psychological elements related to cognition, emotion, and behavior. Cognitive processes are necessary for recognizing and understanding comic content, affective processes concerning experiencing amusement, and behavior related to the expression of emotions [38]. Cermak et al. [39] measured humor and found that people with chronic AUD chose endings more unrelated to the main topic (even though they did not differ from healthy controls (HC) in terms of choosing the correct answers), which could indicate impairments in integrating different aspects of a narrative. Uekermann et al. [30] presented jokes with four different alternative endings (correct funny punchline, a slapstick alternative, and logical and illogical alternatives) to individuals with AUD and had them assign funniness ratings. The results showed that people with AUD chose a lower number of correct punchlines and a higher number of slapstick endings and logical alternatives than HC. Moreover, they found different endings (correct, slapstick, and illogical alternatives) less funny. These findings revealed impaired affective and cognitive aspects of humor processing in people with AUD. Another study revealed cognitive impairment based on speech content analysis in this group [40].

To our knowledge, no study to date has used the RHLB battery to assess a wide range of language and communication abilities in AUD patients. Moreover, most previous studies did not control for the effects of depression and executive functions when comparing some aspects of HOLF (e.g., prosody or humor) between AUD patients and HC. Controlling these variables is important because other studies have demonstrated the existence of a relationship between them [30,41,42], and patients with AUD and depression compared to healthy controls have worse results in cognitive tasks [43]. Moreover, depressive disorder is the most common co-occurring psychiatric disorder among people with AUD [44]. The results of some studies in this area remain contradictory and inconclusive [27]. Given the limitations of the above findings, the aim of this study was to compare AUD patients with HC in terms of skills concerning inference from context, lexical-semantic processes, comprehension of humor and metaphors, understanding emotional and linguistic prosody, ability to withhold inappropriate comments, and understanding the discourse, while controlling for depression, speed of processing, and executive functions. The results of this study may be important for diagnostic and therapeutic processes in this clinical group.

## 2. Materials and Methods

### 2.1. Participants

A total of 75 participants took part in the study: 31 (23 males and 8 females) patients with AUD and 44 HC (27 males and 17 females). All subjects were native speakers of Polish. Patients with AUD were selected based on medical history, consultation with psychiatrists, and a clinical interview based on the ICD-10 (WHO [45]) with the patient, which was performed before the neuropsychological assessment. Inclusion criteria were being aged between 18 and 60, comprehension of the study procedures, and written consent to take part in the study. Exclusion criteria for both groups were psychiatric and neurological conditions, severe somatic diseases, brain injury, dementia, and substance use disorders (other than AUD in the AUD patients). The study was conducted in psychiatric and therapeutic clinics. The study was approved by the Ethics Committee for Research Projects at the Institute of Psychology of the University of Gdańsk (6/2015).

### 2.2. Neuropsychological Assessment

The Polish version of the Right Hemisphere Language Battery (RHLB [4]) was used to measure different aspects of HOLF. The battery consists of 11 subtests: Inferential Meaning, Lexical-Semantic, Humor, Commentary, Written Metaphor, Picture Metaphor, Written Metaphor Explanation, Picture Metaphor Explanation, Emotional Prosody, Linguistic Prosody, and Discourse Analysis.

In the Inference Test, the respondent answered questions about the information hidden in short narrative or conversational stories (scoring is between 0 to 16). The correct performance of the Lexical-Semantic Test requires understanding a heard word and indicating the corresponding picture (scoring is between 0 to 13). There are also 5 additional distraction pictures: 2 semantic, 1 functional, 1 phonological, and 1 visual control. The Humor Test consists of choosing an appropriate and humorous ending for a given story; there are 2 wrong endings: a neutral ending (adequate to the story, but not funny) and a surprising ending (not consistent with the content of the story; scoring was between 0 to 10). The Commentary Test is the sum of points scored for comments made while performing the Inference Test and Humor Test (scoring is between 0 to 14). In the Written Metaphor test, the respondent is asked to identify a metaphorical answer from among 3 answer variants: the correct metaphorical answer, a literal answer, and an inappropriate meaning (scoring is between 0 to 10). In the Written Metaphor Explanation test, the subject gives their own interpretation of the metaphor from the previous test (scoring is between 0 to 10). The Picture Metaphor test requires indicating from among 4 pictures the one that reflects the correct meaning of the metaphor (scoring is between 0 to 10). The incorrect pictures consist of 1 showing the literal meaning and 2 other controls that correspond to some part of the sentence. In the Picture Metaphor Explanation test, the respondent again explains the metaphors in their own words (scoring is between 0 to 10). The Emotional Prosody Test is intended to assess the ability to understand emotional intonation (happiness, anger, sadness; scoring is between 0 to 16). There are 16 nonsense phrases played from a recording; after each, participants are asked to indicate the appropriate tone of expression. The Linguistic Prosody test consists of a similar procedure. However, the subject hears recorded phrases with intonations expressing a statement, question, and an order (scoring is between 0 to 16). The last test is Discourse Analysis, which comprises 16 rating scales that assess the ability of the respondent to interact with another person and make a conversation (scoring is between 0 to 60).

In the Polish population of HC and patients with right hemisphere damage, the psychometric properties of RHLB were validated by Łojek [4]. The battery has acceptable reliability (test-retest, no significant differences between 2 time-points and significant Pearson correlation coefficient between 0.43 and 1.00 for most indices) and diagnostic validity (significant differences between patients with brain damage and HC in all indices). The Polish version of Color Trails Test (CTT) by Łojek and Stańczak [46] measured speed of processing and executive functions as co-variates. This culture-free tool consists of 2 parts: (a) CTT 1, in which the respondent connects a series of 25 numbered circles using a pencil and paper and (b) CTT 2, in which they connect numbered circles from 1 to 25 that alternate from pink to yellow. We used time taken to complete CTT 1 as a measure of the speed of processing and time taken to complete CTT2 as a measure of executive functions.

### 2.3. Clinical Assessment

A structured clinical interview was conducted with each participant. It included demographic data such as age, gender, years of education, and clinical data regarding AUD duration, length of stay in the treatment department, number of times in the therapeutic department, and number of times in the withdrawal department. Data were verified based on the patient’s medical history. The severity of depressive symptoms (occurring during the last 2 weeks) was assessed with the Polish version of the Patient Health Questionnaire-9 (PHQ-9 [47,48]. This tool consists of 9 questions, and answers are scored on a scale from 0 (low severity of the symptom in the last 2 weeks) to 3 (very frequent occurrence of the symptom in the last 2 weeks). The maximum number of points that can be obtained is 27, which indicates the greatest intensity of depression.

### 2.4. Statistical Analysis

Statistical analysis of the results was conducted using the IBM Statistical Package for the Social Sciences 26 (IBM Corp., Redmont, VA, USA). Continuous variables were presented as means (*M*) and standard deviations (*SD*). G-Power 3.1.9.7 software was used for sample size the calculation for the two-sample *t*-test. We assumed a power of 0.90 and α = 0.05 to detect a large effect size (0.80) for differences in HOLF between the 2 groups [49]. According to this calculation, the minimum number of participants was 28 for each of the subgroups, thus 56 participants in total. Based on previous findings (where different measures were used to assess HOLF and executive functions) we assumpted the large effect size of differences between groups in RHLB [33,34,35,50] and CTT [51]. The normality of the distributions was tested with the Shapiro–Wilk test as well as skewness and kurtosis values. The differences between the 2 groups were examined with independent sample Student’s *t*-tests. For significant differences in correct and incorrect RHLB answers between the 2 groups, an analysis of covariance (ANCOVA) was performed to control for the effect of depression (results on PHQ-9), and speed of processing and executive functions (results on both parts of the CTT), because these variables might be related to HOLF [30,41]. Cohen’s *d* or ɳ^2^ [52] was used to determine the magnitude of effect sizes. Holm–Bonferroni *p*-value correction was used for all statistical analyses due to multiple comparisons. The alpha criterion level was assumed at 0.05 and statistical power above 1-ꞵ = 0.80 for all statistical analyses [53]. We also interpreted the effect of the difference as a statistical trend on level (0.05 < *p* < 0.10), which was recommended in the literature of biomedical sciences [54].

## 3. Results

### 3.1. Demographic, Clinical, and Psychological Characteristics

Demographic, clinical, and psychological characteristics are presented in Table 1. The groups did not differ in the proportion of males and females, age, or years of education. However, compared to HC, patients with AUD (after Holm–Bonferroni *p*-value correction) had higher scores on PHQ-9 (*p* < 0.001) and longer reaction times on CTT 1 (*p* = 0.012) and CTT 2 (*p* = 0.008).

### 3.2. Group Differences in Correct Answers on the RHLB

As shown in Table 2, compared to HC, patients with AUD (after Holm’s *p*-value correction due to multiple comparisons) had worse results in terms of correct answers on 9 RHLB tests: Inferential Meaning (*p* < 0.001), Lexical-Semantic (*p* = 0.006), Commentary (*p* = 0.044), Picture Metaphor (*p* < 0.001), Picture Metaphor Explanation (*p* = 0.044), Written Metaphor Explanation (*p* < 0.001), Emotional Prosody (*p* = 0.025), Linguistic Prosody (*p* < 0.001), and Discourse Analysis (*p* < 0.001). Differences between groups on the Inferential Meaning Test, Lexical-Semantic Test, Picture Metaphor Test, Picture Metaphor Explanation, Written Metaphor Explanation, Linguistic Prosody Test, and Discourse Analysis (0.001 > *p* < 0.05) remained significant after co-varying for depression, speed of processing, and executive functions. In the case of results on the Commentary Test and Emotional Prosody Test, group differences were at the level of marginally significant (*p* < 0.01). The effect sizes (ɳ^2^) of the analyzed indices were found to be 0.04–0.21, indicating small to large effect sizes.

### 3.3. Group Differences in Incorrect Answers on the RHLB

As shown in Table 3, compared to HC, patients with AUD (after Holm’s *p*-value correction due to multiple comparisons) had worse results for two incorrect answers on the Picture Metaphor Test—Literal Answers (*p* < 0.001) and Written Metaphor Explanation—Abstract Incorrect (*p* < 0.001). Differences in both indices (0.01 > *p* < 0.001) remained significant after co-varying for depression, speed of processing, and executive functions. The effect sizes (ɳ^2^) of the analyzed indices were found to be 0.14–0.21, indicating large effect sizes.

## 4. Discussion

The aim of the present study was to investigate differences in HOLF between patients with AUD and HC. The results revealed disturbances in the ability to infer from context and understanding of lexical-semantic information (understanding of heard words and their visual analogs) in patients with AUD. They had reduced capacity for abstract thinking based on visual-spatial analysis of information (picture metaphors) and problems with explaining metaphors. AUD patients had a greater tendency to make spontaneous inappropriate comments during the test. Impaired understanding of emotional and linguistic prosody was also observed. Moreover, patients with AUD were less effective at interpersonal communication. However, both groups had similar levels of comprehension of humor and written metaphor, represented by abstract thinking based on the processing of complex language material.

In our study, we expected that patients with AUD would have decreased HOLF in comparison to HC, as measured by the RHLB. These assumptions were confirmed and are in-line with some previous research that studied selected aspects of language, communication, and social cognition [27,40]. In previous studies, affective prosody impairments and difficulties in emotional decoding have been quite extensively researched in patients with AUD [28,32,33,55]. Monnot et al. [56] suggested that, as a result of changes in the brain, people with AUD have greater difficulty understanding the emotional valence of speech, which can impact personal relationships in different situations as well as the effectiveness of therapeutic interventions. Our research has shown not only emotional prosody disorders but also greater dysfunctions in linguistic prosody, which have not yet been studied in this group (confirmed by differences in effect size). Patients with AUD had difficulties understanding the mode of sentences (affirmative, questioning, imperative), which can significantly limit communication between people.

AUD patients in our study also found it difficult to make inferences based on the texts they read. The stories were narrative or conversational. In the study of Cermak et al. [39], people with AUD showed reduced ability to draw inferences, which was in line with our results. On complex and cognitively demanding inference tasks that require the integration of information contained in sentences, patients with AUD had more difficulties than HC. This suggests that AUD may interfere with the ability to make inferences. These deficits may result from impairments in working memory often found in patients with AUD [57] because story comprehension is related to memory processes and activation of information relevant to the currently processed word. It is also associated with the inhibition of irrelevant information, leaving only that which may aid comprehension of the concrete utterances [58]. Our results may be explained in light of Kintsch’s Construction-Integration model, which suggests that the currently processed word or clause automatically and indiscriminately activates information concerning this word, relatively ignorant of context [59].

Difficulties with comprehending narratives may be related to deficits interpreting lengthy, grammatically complex sentences and problems combining semantic and syntactic information [9]. It should be noted that the results of our research also showed that AUD patients had difficulties with lexical-semantic processing and visual-spatial analysis, in which they had to match a word to a picture. In other words, patients present a poorer ability to link words with their visual analogs. This might be due to the difficulty of transforming verbal information into visuals. After verbal instructions, the visual mental images might become somewhat intrusive in the visual task [60]. Additionally, lexical and semantic disorders may be associated with lower inference ability and cause problems understanding more linguistically complex texts [61].

Furthermore, we observed that AUD patients had difficulties comprehending conventional metaphors (which have not been studied in this group). These are a type of figurative expression that requires knowledge of semantic and syntactic rules, non-linguistic skills, and recollection of meaning [62]. Patients had problems identifying the appropriate picture illustrating the meaning of a metaphor and explaining metaphors in their own words. Additionally, they gave more incorrect answers (literal answers and abstract incorrect) than did HC. Visuospatial material is frequently difficult to put into words, therefore it requires the ability to process information in new and creative ways [39] and, as mentioned above, transforming verbal information into visual information can be difficult. Moreover, the assessed group did not differ in the selection of the meaning of written metaphors from three possible answers. This could have been caused by the construction and difficulty of the test. Patients had to choose one correct answer from three others, and this task might have been too easy compared to other metaphor processing tasks. There are no studies in this area in people with substance use disorders, thus a comparison is not possible.

Furthermore, our study showed that AUD patients do not differ from healthy controls in understanding humor. There is very little data about the processing of these functions and they are inconclusive. Some results are in line with our study but were obtained with other measurement tools [39]. Although the patients chose the correct answers, they also tended to choose endings more unrelated to the main topic. This may indicate impairments in integrating different aspects of a narrative but not reflect an inability to interpret humorous information [39]. Other data indicate difficulties in understanding irony in people with AUD [28]. However, a study by Uekermann et al. [30] revealed impairment of cognitive and affective components of humor processing in people with AUD. The inconsistency between the results of their study and ours is probably due to the use of different methods to assess humor processing. The authors of this study used more complex and difficult tasks. Further research is needed in this area.

Patients with AUD presented a poorer ability to withhold inappropriate comments during the research procedure. Compared to the control group, they expressed more spontaneous comments related to the study situation and tests. Unfortunately, results concerning withholding inappropriate comments cannot be compared with other studies. These difficulties may be due to increased impulsivity observed in individuals with AUD and impairment of executive function [51,63]. In our study, patients with AUD had a slower speed of processing and executive functions as measured by the CTT. Thus, our study controlled these variables when comparing HOLF between these two groups. Moreover, our study controlled the severity of depressive symptoms, as some studies indicate that they are associated with language and communication functions [36,64,65,66]. Despite the fact that our research confirmed executive dysfunctions and greater severity of depressive symptoms in patients with AUD, they did not change most of the results showing difficulties in HOLF in this clinical group.

Difficulties understanding the meaning of individual words, information implied in text, abstract, metaphorical content, and prosody may impede comprehension and production of discourse, in which linguistic elements must be integrated with non-verbal cues and contextual information [4]. Disturbances of language and communication in patients with AUD may impact various spheres of everyday life and may negatively influence social, private, and professional functioning. Misunderstanding intentions and emotions in conversations and problems with inference reduce the quality of a conversation and may lead to conflicts between interlocutors. It is, therefore, crucial to making neuropsychological assessments of these dysfunctions in AUD patients; the RHLB is a standardized tool, which could be used to do so. The potential role of these deficits should be considered in the therapy of AUD, and specific treatment programs need to be developed. In addition, a detailed analysis of impairments in higher-order language functions in people with AUD may be important for clinical diagnostics.

However, this study has some limitations. First, the group was small, thus it is worth conducting similar research on a larger number of people. In addition, craving scales or consumption scales were not used to quantify the severity of the substance use disorder. It would be worth measuring the quantity of alcohol consumed. Furthermore, we did not control for intelligence. However, we used executive functions as a covariate in the statistical analysis of group differences that may be related to HOLF. It is worth noting that the directionality of the effects cannot be established based on the current study. There is substantial evidence, as with many other substance use disorders, for genetic predispositions to AUD [67], and it is possible that AUD itself might not be the causative factor in the discrepancies found in the current study. Moreover, in our study, we did not compare males and females in HOLF. Thus, this should be explored in future studies. Finally, the reliability and validity of the RHLB in patients with AUD need to be investigated. Despite these limitations, our results suggest that communication and language impairments are present in patients with AUD and can be assessed by the RHLB. Further research on these functions with a larger sample size may provide a better understanding of them in this group of people.

## 5. Conclusions

In summary, our study found that patients with AUD have difficulties with different aspects of HOLF: the ability to infer from context, understanding of lexical-semantic information, comprehension of metaphors, prosody, and production of discourse. Moreover, patients had higher levels of depression, executive dysfunction, and a slower speed of processing. The differences in most HOLF remained significant after co-varying these control variables. These disturbances may impact various spheres of everyday life. Further studies should verify the results of our research.

## Figures and Tables

**Table 1:** Descriptive demographic, clinical, and psychological characteristics of patients with alcohol use disorder (AUD) and healthy controls.

	Patients with AUD *n* = 31	Healthy Controls *n* = 44	χ^2^/*t*	Effect Size (*d*)
Gender: males/females	23/8	27/17	1.35 ^a^	–
Age in years: *M* (*SD*)	37.94 (8.88)	35.39 (10.11)	−1.13 ^b^	–
Years of education: *M* (*SD*)	11.55 (2.42)	11.71 (1.81)	0.32 ^b^	–
AUD duration in years: *M* (*SD*)	13.10 (6.37)		min–max: 6–24	
Length of stay in the treatment department: *M* (*SD*)	2.20 (1.69)		min–max: 1–5	
Number of times in the therapeutic department: *M* (*SD*)	1.40 (1.58)		min–max: 0–5	
Number of times in the withdrawal department: *M* (*SD*)	2.50 (2.22)		min–max: 0–7	
Depression in PHQ-9: *M* (*SD*)	10.26 (5.01)	4.61 (2.99)	−5.61 ^b,^***	1.30
CTT 1 (time reaction): *M* (*SD*)	49.94 (17.35)	39.27 (10.79)	−3.03 ^b,^*	0.70
CTT 2 (time reaction): *M* (*SD*)	96.71 (30.94)	75.59 (21.89)	−3.27 ^b,^**	0.76

*M* = mean; *SD* = standard deviation; CTT = Color Trails Test; PHQ-9 = Patient Health Questionnaire-9 items version; min = minimum; max = maximum. ^a^ Chi-squared test. ^b^ Student’s *t* test (Holm–Bonferroni *p*-value correction for multiple comparisons was used). * *p* < 0.05. ** *p* < 0.01. *** *p* < 0.001.

**Table 2:** Differences in correct answers in Right Hemisphere Language Battery (RHLB) between patients with alcohol use disorder (AUD) and healthy controls.

	Patients with AUD *M* (*SD*) *n* = 31	Healthy Controls *M* (*SD*) *n* = 44	*t*	Effect Size (*d*)	*F*	Effect Size (ɳ^2^)
Inferential meaning test	12.58 (1.77)	14.32 (1.44)	4.68 ^a,^***	1.09	18.71 ^b,^***	0.21
Lexical-semantic test	11.97 (1.11)	12.75 (0.44)	3.73 ^a,^**	0.87	7.81 ^b,^**	0.10
Humour test	8.77 (2.05)	9.30 (1.19)	1.28 ^a^	0.30	–	–
Commentary test	1.55 (2.08)	0.48 (0.93)	−2.69 ^a,^*	0.63	3.16 ^b,^^	0.04
Picture metaphor test	6.16 (2.84)	8.77 (1.48)	4.69 ^a,^***	1.09	15.94 ^b,^***	0.19
Written metaphor test	9.58 (0.67)	9.71 (0.63)	0.82 ^a^	0.19	–	–
Picture metaphor explanation	8.55 (1.50)	9.34 (0.86)	2.65 ^a,^*	0.62	4.83 ^b,^*	0.06
Written metaphor explanation	8.07 (1.59)	9.41 (0.84)	4.30 ^a,^***	1.00	10.62 ^b,^**	0.13
Emotional prosody test	13.29 (2.08)	14.52 (1.23)	2.95 ^a,^*	0.69	3.30 ^b,^^	0.05
Linguistic prosody test	12.97 (2.21)	14.93 (1.36)	4.76 ^a,^***	1.11	13.14 ^b,^**	0.16
Discourse analysis	55.61 (3.99)	59.07 (1.39)	4.63 ^a,^***	1.08	4.59 ^b,^*	0.06

*M* = mean; *SD* = standard deviation. ^a^ student’s *t* test (Holm–Bonferroni *p*-value correction for multiple comparisons was used). ^b^ Analysis of covariance (ANCOVA) to control the effect of depression, speed of processing, and executive functions. ^ *p* < 0.10. * *p* < 0.05. ** *p* < 0.01. *** *p* < 0.001.

**Table 3:** Differences in incorrect answers in Right Hemisphere Language Battery (RHLB) between patients with alcohol use disorder (AUD) and healthy controls.

	Patients with AUD *M* (*SD*) *n* = 31	Healthy Controls *M* (*SD*) *n* = 44	*t*	Effect Size (*d*)	*F*	Effect Size (ɳ^2^)
Lexical-semantic test:						
Sematic incorrect	0.03 (0.18)	0.07 (0.26)	0.68 ^a^	0.16	–	–
Action incorrect	0.23 (0.50)	0.00 (0.00)	–	–	–	–
Phonetic incorrect	0.26 (0.44)	0.05 (0.21)	−2.47 ^a^	0.57	–	–
Graphic incorrect	0.52 (0.81)	0.14 (0.35)	−2.45 ^a^	0.57	–	–
Humour test:						
Abstract incorrect	0.55 (1.41)	0.21 (0.55)	−1.29 ^a^	0.30	–	–
Neutral incorrect	0.68 (1.38)	0.50 (0.85)	−0.69 ^a^	0.16	–	–
Picture metaphor test:						
Literal answers	2.65 (2.07)	0.64 (0.99)	−5.01 ^a,^***	1.16	18.99 ^b,^***	0.21
Inappropriate meaning	1.19 (1.64)	0.59 (0.99)	−1.82 ^a^	0.42	–	–
Written metaphor test:						
Literal answers	0.07 (0.25)	0.00 (0.00)	–	–	–	–
Inappropriate meaning	0.36 (0.61)	0.27 (0.58)	−0.59 ^a^	0.14	–	–
Picture metaphor explanation:						
Literal incorrect	0.29 (0.53)	0.11 (0.32)	−1.66 ^a^	0.39	–	–
Abstract incorrect	1.16 (1.34)	0.55 (0.76)	−2.30 ^a^	0.53	–	–
Lack of answer	0.03 (0.18)	0.00 (0.00)	–	–	–	–
Written metaphor explanation:						
Literal incorrect	0.10 (0.30)	0.05 (0.21)	−0.87 ^a^	0.20	–	–
Abstract incorrect	1.84 (1.49)	0.55 (0.76)	−4.45 ^a,^***	1.03	10.88 ^b,^**	0.14
Lack of answer	0.00 (0.00)	0.00 (0.00)	–	–	–	–

*M* = mean; *SD* = standard deviation. ^a^ Student’s *t* test (Holm–Bonferroni *p*-value correction for multiple comparisons was used). ^b^ Analysis of covariance (ANCOVA) to control the effect of depression, speed of processing, and executive functions. ** *p* < 0.01. *** *p* < 0.001.

## Data Availability

Materials of the review reported here are available from the corresponding author on reasonable request.
